# Analysis of intracranial haemorrhage following tozinameran (BNT162b2, Pfizer-BioNTech)

**DOI:** 10.1186/s40545-021-00354-3

**Published:** 2021-08-04

**Authors:** Yohhei Hamada

**Affiliations:** Institute for Global Health, University College London, 3rd floor, Institute of Child Health, 30 Guilford Street, London, WC1N 1EH UK

Shimazawa et al. suggested a potential causal link between tozinameran and intracranial haemorrhage (ICH) based on what they claimed as incompatibility with national statistics [1]. However, their analysis is flawed. They claim that there was an imbalance in the number of fatal ICH, as there were four such cases in women compared to none in men when the national death rate by ICH is comparable between the sexes. However, they failed to recognize the sex imbalance in the number of health workers in Japan. Their analysis should have accounted for the difference in the denominator. While there are no data on the number of health care workers vaccinated by sex, the number of female health workers from the latest national data is three times higher than that of male [2, 3].

Second, the authors pointed out that the death rate by ICH in the national statistics was 25% lower than that by ischemic stroke. However, they ignored the cause-specific death rate by age group. While fatal ICH was reported in people younger than 80 years, the death rate from ICH in the national statistics is comparable or higher than that by ischemic stroke in those age groups in women (Table 1) [4]. While I agree with the authors that continuous monitoring is recommended to detect signals of rare events, their claim that “our analysis reveals a disproportionately high incidence of death by ICH in Japanese women who received tozinameran, suggesting a potential association of ICH with the vaccine” is unfounded and misleading.

**Table 1:** Death rate by type of stroke in women aged between 20 and 79 years, per 100,000 population

Cause of deaths	Age group											
	20–24	25–29	30–34	35–39	40–44	45–49	50–54	55–59	60–64	65–69	70–74	75–79
Intracranial haemorrhage	0.2	0.1	0.4	0.9	2	3.5	5.2	6.7	8.6	13.6	21.4	42
Ischemic stroke	0	0	0.1	0.1	0.2	0.6	0.8	1.5	3.1	7.4	16.9	45.4
Subarachnoid haemorrhage	0.1	0.3	0.7	1.3	2.4	4.7	6.3	7.1	8.8	11.3	16.1	25.2

## References

[1] Shimazawa R, Ikeda M (2021). Potential adverse events in Japanese women who received tozinameran (BNT162b2, Pfizer-BioNTech). J Pharm Policy Pract.

[2] Ministry of Health, Labour and Welfare. Overview of statistics on doctors, dentists and pharmacists in 2018. December 19, 2019. https://www.mhlw.go.jp/toukei/saikin/hw/ishi/18/index.html. Accessed 25 June 2021.

[3] Ministry of Health, Labour and Welfare. Summary of health administration report examples (medical personnel in employment) in 2018. September 4, 2019. https://www.mhlw.go.jp/toukei/saikin/hw/eisei/18/. Accessed 25 June 2021.

[4] Ministry of Health Labour and Welfare. Death rates (per 100,000 population) by causes (the condensed list of causes of death for Japan), sex and age (5-year age groups): Japan, 2019. https://www.e-stat.go.jp/en/stat-search/files?page=1&layout=datalist&toukei=00450011&tstat=000001028897&cycle=7&year=20190&month=0&tclass1=000001053058&tclass2=000001053061&tclass3=000001053065&stat_infid=000031982755&result_back=1&tclass4val=0. Accessed 25 June 2021.

